# Breast cancer detection: Shallow convolutional neural network against deep convolutional neural networks based approach

**DOI:** 10.3389/fgene.2022.1097207

**Published:** 2023-01-04

**Authors:** Himanish Shekhar Das, Akalpita Das, Anupal Neog, Saurav Mallik, Kangkana Bora, Zhongming Zhao

**Affiliations:** ^1^ Department of Computer Science and Information Technology, Cotton University, Guwahati, India; ^2^ Department of Computer Science and Engineering, GIMT Guwahati, Guwahati, India; ^3^ Department of AI and Machine Learning COE, IQVIA, Bengaluru, Karnataka, India; ^4^ Center for Precision Health, School of Biomedical Informatics, The University of Texas Health Science Center at Houston, Houston, TX, United States; ^5^ Department of Environmental Health, Harvard T. H. Chan School of Public Health, Boston, MA, United States; ^6^ Department of Pharmacology and Toxicology, University of Arizona, Tucson, AZ, United States; ^7^ Department of Pathology and Laboratory Medicine, McGovern Medical School, The University of Texas Health Science Center at Houston, Houston, TX, United States

**Keywords:** breast cancer, medical imaging, deep learning, convolutional neural networks, transfer learning

## Abstract

**Introduction:** Of all the cancers that afflict women, breast cancer (BC) has the second-highest mortality rate, and it is also believed to be the primary cause of the high death rate. Breast cancer is the most common cancer that affects women globally. There are two types of breast tumors: benign (less harmful and unlikely to become breast cancer) and malignant (which are very dangerous and might result in aberrant cells that could result in cancer).

**Methods:** To find breast abnormalities like masses and micro-calcifications, competent and educated radiologists often examine mammographic images. This study focuses on computer-aided diagnosis to help radiologists make more precise diagnoses of breast cancer. This study aims to compare and examine the performance of the proposed shallow convolutional neural network architecture having different specifications against pre-trained deep convolutional neural network architectures trained on mammography images. Mammogram images are pre-processed in this study's initial attempt to carry out the automatic identification of BC. Thereafter, three different types of shallow convolutional neural networks with representational differences are then fed with the resulting data. In the second method, transfer learning via fine-tuning is used to feed the same collection of images into pre-trained convolutional neural networks VGG19, ResNet50, MobileNet-v2, Inception-v3, Xception, and Inception-ResNet-v2.

**Results:** In our experiment with two datasets, the accuracy for the CBIS-DDSM and INbreast datasets are 80.4%, 89.2%, and 87.8%, 95.1% respectively.

**Discussion:** It can be concluded from the experimental findings that the deep network-based approach with precise tuning outperforms all other state-of-the-art techniques in experiments on both datasets.

## 1 Introduction

Cancer affects several body organs, including the lungs, pancreas, blood, and breasts, making it more than simply a typical sickness. These cancer forms are similar in some ways, yet they differ in their modes of development and dissemination. Breast cancer affects women mostly and is thought to be the second-deadliest illness in women, according to the International Agency for Research on Cancer (IARC) study released by the World Health Organization (WHO) in 2012 (Boyle and Levin, 2008). According to the Indian Council of Medical Research (ICMR), 2018 in India, breast cancer accounts for the biggest percentage of malignancies and is the second most prevalent disease overall that affects women, with 87,090 fatalities on average (Mathur et al., 2020). With an anticipated 2.3 million new cases, or 11.7% of all cancer cases, it now overtook lung cancer as the most common kind of cancer worldwide in 2020 (Sung et al., 2021). Cancer develops in the human body as a result of the tumor cells’ aberrant development and invasion of the surrounding tissues. Tumors are often divided into benign and malignant categories. In contrast to malignant tumor cells, which are thought to be cancerous, benign tumor cells are not carcinogenic. The benign tumor’s cells proliferate only in that area of the body and are unable to invade nearby tissues to spread. On the other hand, malignant tumor cells can develop out of control, infiltrate nearby tissue, and eventually spread to different areas of the body (Prusty et al., 2022). To diagnose breast cancer, a variety of screening techniques are utilized, although mammography is by far the most effective. In mammography, several perspectives, such as the craniocaudal (CC) and medial-lateral oblique (MLO), are utilized to better comprehend the breast abnormalities that are present. Radiologists employ any of these MLO or CC views to examine breast lesion indicators such as masses and micro-calcifications to differentiate between benign, normal, and malignant classifications of breast abnormalities (Bick, 2014). It takes a lot of time and knowledge from a radiologist with extensive training and experience to interpret mammographic images (Hubbard et al., 2011). These problems have led to an increase in the need for computer-aided diagnosis and detection (CAD) technologies, which automate medical image processing (Das et al., 2022).

Deep learning has made significant strides in research over the past 10 years (Das et al., 2021), and the subject of healthcare is no exception (Das et al., 2020). In the research on breast cancer, which produced encouraging findings as well, several deep architectures (Hamidinekoo et al., 2018) are investigated and effectively applied. The use of computer-aided diagnostics to diagnose breast cancer is still being studied by a sizable number of researchers. In a hybrid Convolutional Neural Network (CNN) approach presented by Arevalo et al. (2016), handmade image-based features are learned using supervised learning techniques. Huynh et al. (2016) used transfer learning to the pre-trained AlexNet model for the classification of mammographic tumors without fine-tuning, and the Support Vector Machine (SVM) technique is utilized for classification at the back end. Recent advances in deep learning technology have the potential to improve the standard of treatment in the healthcare sector (Carneiro et al., 2015). Fine-tuned ImageNet, a pre-trained CNN, to discriminate between masses and micro-calcification. The Breast Imaging-Reporting and Data System (BI-RADS) score is particularly helpful for identifying the kind of breast cancer. In their study, the authors used the BI-RADS score to distinguish between various forms of breast cancer. For the classification of mammographic images, Levy and Jain (2016) used transfer learning on two pre-trained models, such as AlexNet and GoogleNet. AlexNet was found to have the greatest results when they compared the two networks. Ting et al. (2019) created a brand-new network named Convolutional Neural Network Improvement for Breast Cancer Classification network (CNNI-BCC). The proposed model was created from scratch and trained for BC classification. The experiment used the MIAS database, and the model was provided with the area of interest (ROIs) identified using a one-shot detector. After training with all CNN, (Rampun et al., 2018) used the three top-performing model predictions in an ensemble and modified version of the AlexNet model. Tsochatzidis et al. (2019) conducted a thorough analysis of several designs, including Inception-BN (v2) from scratch, GoogleNet, ResNet50, ResNet101, ResNet152, AlexNet, VGG16, and VGG19. Both the Digital Database for Screening Mammography (DDSM-400) and Curated Breast Imaging-Digital Database for Screening Mammography (CBIS-DDSM) datasets might use some fine-tuning. Arora et al. (2020) proposed a high ensemble transfer learning model to differentiate between benign and malignant tumors. A neural network classifier is then used to do auto-feature extraction. In the literature, several deep learning models have been proposed. To identify and classify benign and malignant lesions included in digital mammography images, authors (Duggento et al., 2019) employed the CNN model. Deep Convolution Neural Network and AlexNet were used by (Ragab et al., 2019) for feature extraction and classification of mammographic images from the DDSM dataset. Another deep CNN was used by Ting et al. (2019) to classify BC-lesion from the MIAS dataset. BreastNet is another CNN-based deep learning model that has been put up by authors (Toğaçar et al., 2020) and beats AlexNet, VGG-16, and VGG-19. The authors (Wang et al., 2020) have also developed bilateral residual GANs (BR-GANs), which are based on the cycle GAN idea, for the job of segmenting mammograms for the INbreast dataset. Mohiyuddin et al. (2022) have suggested modified YOLOv5 for the identification and categorization of breast tumors of the DDSM dataset. Al-Antari et al. (2018) are using AlexNet, a second CNN-based classifier, in addition to YOLO for multiclass classification for the INbreast dataset. U-Net is renowned for its effectiveness in the segmentation of medical images. For the segmentation challenge, Zeiser et al. (2020) applied an architecture based on U-Net to a variety of publicly and privately accessible datasets. The segmentation of masses on mammography images is the focus of their work. Authors (Abdelhafiz et al., 2020) have also presented the Vanilla U-Net, another form of U-Net, for segmenting mass in mammography images obtained from three separate publicly accessible datasets. For breast density segmentation, Saffari et al. (2020) suggested a conditional form of the GAN (cGAN) and U-Net-based cGAN-UNET model. Later, CNN was applied for classification purposes for the INbreast dataset. The efficiency of transfer learning for breast cancer categorization was demonstrated by Rahman et al. (2020). For the DDSM dataset, the authors tested the transfer learning-based models. Similar transfer learning principles were applied by Saber et al. (2021) for the categorization of breast cancer using a variety of pre-trained deep learning models, including Inception V3, ResNet50, Visual Geometry Group networks (VGG)-19, VGG-16, and Inception-V2. Another method for classifying breast cancer using multi-DCNNs was proposed by Ragab et al. (2021), who similarly employed the idea of transfer learning for feature extraction and SVM for final classification on the MIAS-DDSM datasets. Lotter et al.’s annotation-efficient deep learning strategy for mammograms and digital breast tomosynthesis image-based breast cancer diagnosis has been proposed by Lotter et al. (2021). Malebary and Hashmi. (2021) suggested an ensemble-based technique for classifying breast masses. In the proposed study, they have employed, pre-trained CNN and RNN-LSTM-based deep learning models to extract both the low-level and high-level features. Finally, the classification was completed by combining the random forest approach with high gradient boosting. Deep learning techniques based on attention have also proven effective in classifying images. Sun et al. (2020) presented the attention-guided deep learning network known as AU-Net, which segments breast mass using an attention-guided upsampling block. Multi-scale attention-based network MSANet was created by Xu et al. (2021) for the categorization of mammograms for the DDSM dataset. Su et al. (2022) introduced the YOLO-LOGO segmentation model, which combines the YOLO and LOGO architectures with a deep learning technique based on transformers for the identification and segmentation of breast masses for DDSM-INbreast datasets.

The Curated Breast Imaging Subset of the Digital Database for Screening Mammography (CBIS-DDSM) and INbreast datasets are used to train several convolutional neural network architectures in this paper’s effort to construct an automated system employing mammographic images. Investigations are carried out in this study to classify breast cancer into benign and malignant categories. In the first technique, three different types of shallow convolutional neural networks have been trained from scratch; in the second approach, various pre-trained convolutional neural networks have also been tested using transfer learning *via* fine-tuning. Convolutional neural network variations such as VGG19, ResNet50, MobileNet-v2, Inception-v3, Xception, and Inception-ResNet-v2 respectively, have each been taken into consideration for this work. The accuracy of various breast cancer datasets for different algorithms as well as with various proposed strategies is the main focus of prior research studies. However, the major focus of this work is on how various deep-learning models behave for complete mammography images. The following are this paper’s key contributions:

• To compare and analyze the performance of shallow convolutional networks against deep convolutional neural networks.
• To analyze the usefulness of various proposed models while working with only full-mammogram images.
• Impact of transfer learning approach on the pre-trained convolutional neural networks using fine-tuning and regularization techniques.

The organization of the paper is as follows: Section 2 explains the database along with the proposed mechanism. Section 3 explains the experimental results of the proposed approaches, followed by the discussion in Section 4. Finally, the conclusion of the paper is presented in Section 5.

## 2 Materials and methods

This section explains the databases that are used for this study. It also briefly describes the proposed model along with the comparative approach of the shallow network against deep convolutional pre-trained networks with their set parameters.

### 2.1 Database description

#### 2.1.1 CBIS-DDSM

It is a section of the DDSM (Lee et al., 2016) database, which contains 6775 studies altogether. The qualified and experienced radiologists who choose the mammographic images from DDSM represent them in an updated and standardized version of CBIS-DDSM. After lossless decompression, all of the images are transferred to the DICOM format. The segmented region of interest (ROI) for training data is also included in the database along with information on pathologic diagnosis. While the information may be generally categorized based on the sorts of anomalies, such as bulk and calcification, it can also be divided into subclasses based on malignant and benign tumors.

#### 2.1.2 INbreast

The INbreast dataset contains 410 different digital mammographic images from 115 patients. The mammography images were interpreted by experienced radiologists, and following the analysis, the lesions identified by the scans were given a standard score known as BI-RADS (Orel et al., 1999). The six BI-RADS scores represent the various stages of abnormalities that can be found in the breasts; score 0 denotes an inconclusive examination; score 1 suggests no findings; score 2 indicates benign; score 3 denotes probably benign findings; score 4 denotes suspicious findings; score 5 ensures a high probability of malignancy, and score 6 denotes breast cancer. The INbreast dataset is not accessible to the general public, although it may be requested from (INbreast Dataset, 2012).

**FIGURE 1:**
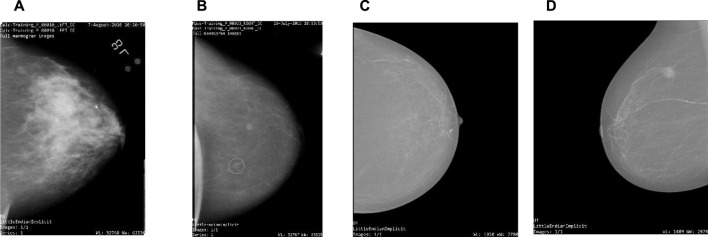
Sample full-mammogram images **(A,B)** from CBIS-DDSM and **(C,D)** from INbreast.

The comprehensive description of both datasets can be found in Table 1. Table 2 gives insights into the distribution of data for full-mammogram images that have been taken into consideration for the proposed work. Figure 1 shows examples of some sample images from both datasets.

**TABLE 1:** Mammography datasets for breast cancer.

Dataset	Type	No. of images	View	Format	Classes
CBIS-DDSM	Digital mammogram (DM)	10,239	MLO/CC	DICOM	Benign and malignant
INbreast	Digital mammogram (DM)	410	MLO/CC	DICOM	Benign and malignant

**TABLE 2:** Distribution of data for full-mammogram images.

Dataset	Type	Train	Test	Total
CBIS-DDSM	Full mammogram	2138	566	2704
INbreast	Full mammogram	328	82	410
Grand total		2466	648	3114

### 2.2 Proposed methodology

**FIGURE 2:**
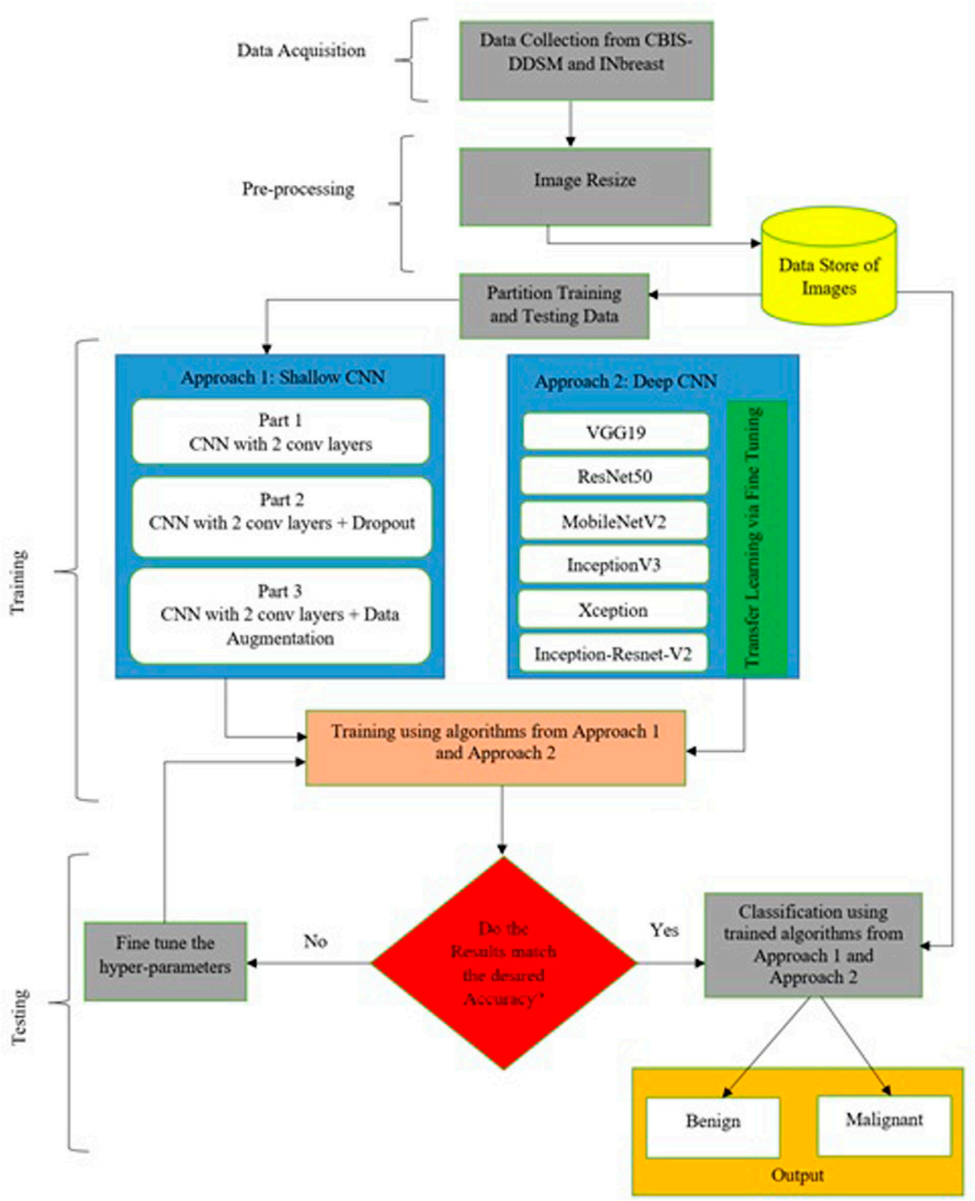
Proposed architecture of classification of breast cancer.

Deep learning architectures’ main contribution is their capacity to autonomously extract low-level to high-level properties (Das and Roy, 2021). CNNs are the best models for identifying detailed properties in images. CNN may learn feature representation automatically as opposed to manually constructed features. Various requirements have been the subject of an extensive investigation in this research. All mammogram images first went through a pre-processing stage in which they were converted from DICOM images to the portable network graphics (PNG) format. The DICOM format is being converted to PNG to prevent loss in image quality. After that, the pixel values between “0” and “1” are normalized to ensure that the higher pixel values have no impact on the investigation’s findings. Thereafter, we have to adjust the labels for the binary classification problem, so that “0” corresponds to “benign” and “1” maps to “malignant.” Then the mammographic images from both datasets are resized to 224 × 224 image size; split the training data are into “training” and “validation” subsets; build Keras generators for training and validation data. The paper consists of two different approaches. A very small CNN with only two convolutional layers has been used for simulation in the first part of the first approach. A dropout layer has been added in the second part to mitigate hard overfitting, and in the third part, data augmentation, another regularization technique, is applied to the previous model to further mitigate overfitting. The second method involves testing a variety of pre-trained CNN networks using a fine-tuning strategy, including VGG19, ResNet50, MobileNet-v2, Inception-v3, Xception, and Inception-ResNet-v2 respectively. Figure 2 provides a concise summary of the suggested methods.

### 2.3 Classification model

Finding the approximate size of the model is the first stage. A model with too many parameters may learn slowly and overfit, whereas a network that is too tiny will not be able to generalize well. Starting with a tiny, naïve model and progressively increasing its size until it begins overfitting while learning is a useful technique to determine the right size. At that point, the model is adaptable enough to suit the training data and may be generalizable to additional data with the right training. Of course, the model may be improved later on to produce better performances by adding new layers, changing the existing ones using regularization techniques, or adjusting the hyperparameters. As mentioned, the study consists of two different approaches. Two convolutional layers interspersed with max-pooling make up a very tiny CNN used in the first part of the first approach. The output is produced at the very end by a single neuron with sigmoid activation following a completely connected layer (binary classification). The loss function is the binary cross-entropy, which is particularly suitable for this kind of problem (binary classification). The optimizer is RMSprop, an adaptive optimization algorithm that is considered quite efficient. Since the validation set is where the model often performs at its best, we keep an eye on how the loss changes there as well throughout training and store the relevant model weights when that loss is minimal. The top objective right now is to lessen the harsh overfitting that was shown in the previous part since it keeps the network from discovering a useful broad set of weights. The second part is identical to the first, with the exception that there is a dropout layer added after the last fully-connected block. Dropout is a potent regularization method that’s frequently used to cut down on overfitting. Every iteration during the training phase involves disregarding a randomly selected portion of the neurons from the preceding layer; this drives the network to identify redundant and alternative representations for the inputs, ultimately improving performance. The dropout rate is set at .5, which is experimentally found to be the ideal number. Data augmentation is used to further regularize the prior model before modifying the architectural arrangement to reduce overfitting. When using data augmentation, specified changes are applied to the original data to produce a greater variety of samples for the network to train on. Image flipping, shifting, rotation, scaling, distortion, and noise injection are common approaches. The Keras image generator has to be reinitialized with the appropriate inputs in order to take use of data augmentation. We apply the augmentation techniques using the following settings: Flipping (horizontal and vertical), Rotation (angle between 0° and 180°), Shear (10 deg), and Scale (.2).

#### 2.3.1 VGG19

VGG19: At the Oxford Robotics Institute, the Visual Geometry Group Network (VGG) was created based on the convolutional neural network architecture (Hemdan et al., 2020), and (Simonyan and Zisserman, 2014) unveiled it. The ImageNet data cluster has shown particularly strong performance from VGGNet. To calibrate 138 million weight parameters, this network underwent more than 370,000 iterations of training on more than 1 million pictures in 1000 classes. Particularly, in the Large-Scale Visual Recognition Challenge, a worldwide image recognition competition, in 2014 (Simonyan and Zisserman, 2014; Sukegawa et al., 2020), VGG19 took first place in a classification and localization competition. VGG11, VGG13, VGG16, and VGG19 are some of the transfer learning networks that make up the VGG. These network architectures all share the trait of having several convolution-layer modules coupled to three complete connection layers (Wan et al., 2021). VGG19 is composed of five components. There are two convolutional layers and one pooling layer in the first and second building blocks, respectively. The third and fourth blocks each contain one pooling layer and four convolutional layers. There are four convolutional layers in the final block. There are also 3 × 3 tiny filters (Arshad et al., 2022).

#### 2.3.2 ResNet50

The most crucial component of ResNet-50 is the residual building block (RBB). The foundation of RBB is the concept of employing shortcut connections to skip whole convolutional layer blocks. In order to avoid the vanishing/exploding gradients problem, these shortcuts help optimize trainable parameters in error backpropagation, which can help to build deeper CNN structures to enhance overall performance for fault detection. Convolutional layers, batch normalizations, the Rectified Linear Unit (ReLU) activation function, and one shortcut make up the RBB (Wen et al., 2020).

#### 2.3.3 MobileNetV2

A widely used CNN-based model for classifying images is called MobileNetV2. The key benefit of adopting the MobileNet architecture is that the model requires far less computing work than the traditional CNN model, making it appropriate for use with mobile devices and personal computers with limited processing power. The MobileNet model is a convolution layer-based simplified structure that can be used to distinguish between the finer details that depend on two controllable characteristics that switch between the parameter’s accuracy and latency efficiently. The MobileNet approach has the benefit of shrinking the network (Srinivasu et al., 2021).

#### 2.3.4 InceptionV3

It takes at least a few days to train the deep neural network model Inception-v3 (Szegedy et al., 2016), which is highly challenging for us to train directly on a low-configured machine. We can use transfer learning to retrain Inception’s final Layer for new categories using lessons from Tensorflow (Abadi et al., 2016). We employ the Inception-v3 (Szegedy et al., 2016) model’s last layer, which is removed while keeping the parameters from the layer before it. The last layer is then retrained. In the final layer, there are as many output nodes as there are categories in the dataset. For instance, the final layer in the original Inception-v3 model contains 1,000 output nodes since the ImageNet dataset has 1,000 classes. During the fine-tuning stage, the model has been tuned as per the number of classes as well as the number of tuned layers.

#### 2.3.5 Xception

Numerous structured models utilizing CNN have been developed as a result of the widespread use of CNNs in computer vision. LeNet-style models (LeCun et al., 1995) were first introduced in 1995, after which several models were developed for use in classification and recognition issues. Inception is one such example. Szagedy et al. developed the Inception architecture, also known as Inception-v1(Szegedy et al., 2015), in 2014. Inception-v2, Inception-v3 (Szegedy et al., 2016), and Inception-ResNet (Szegedy et al., 2017) were later updated. An explanation of the Inception modules may be found in the Xception network (Chollet, 2017) that was employed in this investigation. “Extreme inception” is also where the word Xception originates. A brief overview of Inception will be conducted initially to better understand the Xception design. The item that has to be recognized in object recognition or picture classification may appear little or huge depending on the image. To put it another way, the size of the item may vary across images. It might be challenging to choose the right filter size for the convolution process due to the different object sizes. For an object that seems enormous in the images, a high filter size should be recommended, while a small filter size should be used for little things. The problems caused by objects of different sizes can be resolved, according to the Inception design, by placing several filters of varying sizes at the input. Additionally, it recommends forwarding this module’s output to yet another inception module.

#### 2.3.6 Inception-ResNet-V2

Szegedy’s Inception-Resnet (Szegedy et al., 2017) design combines the Resnet and Inception network backbone systems. The Inception module is a network with good local topology, which enables it to conduct simultaneous convolution or pooling operations on the input image. It does not limit itself to a single convolution kernel, but rather employs all convolution kernels of various sizes simultaneously, merging the output of each convolution to create a more detailed feature map. Benefiting from it may result in improved visual representation (Wu et al., 2017). Kaiming He, submitted Resnet (He et al., 2016), a residual neural network architecture of 152 layers, in the ImageNet competition. The neural network’s shortcut architecture was introduced by him.

### 2.4 Transfer learning

Typically, a large dataset is needed to train the CNN (at least thousands of samples if not available in millions). Due to the restricted time and effort of professionals to provide labeled sample datasets on medical images, it is challenging to use CNN trained from scratch. Methods based on the CNN typically overfit and are unable to extract the image features in good quality when the training dataset is short, as is the situation in this area of medical image analysis. Transfer learning is a technique that allows a CNN to be initially trained on a large-scale labeled image dataset to learn standardized image properties before being used to obtain comparable features from a smaller dataset. It has already been effectively used in many image-processing applications and clinical studies for diseases. For all assessments and comparisons, Table 3 shows the fine tweaking done with the pre-trained models.

**TABLE 3:** Pre-trained CNNs with fine-tuned parameters.

Models	Number of layers added	Number of nodes added	Dropout rate (%)
VGG19 Simonyan and Zisserman, (2014)	3	2048, 1024, 1024	0.5%
ResNet50 Akiba et al. (2017)	2	1024, 512	0.5%
MobileNet-v2 Howard et al. (2017)	2	2048, 1024	0.5%
Inception-v3 Xia et al. (2017)	1	1024	0.6%
Xception Chollet., 2017	2	1024, 512	0.5%
Inception-ResNet-v2 Szegedy et al. (2017)	1	1024	0.7%

## 3 Results and discussion

To detect BC from mammography images, the authors used two distinct methods. The comparison and analysis of the performance of shallow CNN and deep CNN models that have already been trained is the main objective. On the CBIS-DDSM and INbreast datasets, we put the suggested ways into practice and evaluated them to determine how well they performed in comparison to the existing methodologies. These models were produced using the Tensor Flow backend and Keras deep learning framework provided by Google Colab.

### 3.1 Performance metrics for evaluation of classification task

A classification model’s performance assessment parameters are based on the model’s accurate and wrong predictions of test records. For the test dataset for all classes, the confusion matrix provides information on how predicted values compare to real values that may be seen. The confusion matrix includes the four measurements true positive (TP), false positive (FP), true negative (TN), and false negative (FN). These four metrics can be used to assess effective parameters for comparing various categorization systems. Table 4 explains the most used performance metrics based on the confusion matrix. When assessing the effectiveness of our proposed approaches in this study, accuracy, precision, recall, and F1 score were taken into account.

**TABLE 4:** Performance measures for the evaluation.

Measures	Formula	Description
Accuracy Urban et al. (2018), Zhang et al. (2016), Bandyopadhyay et al. (2013), Bedrikovetski et al. (2021)	$\frac{\left|TP\right|+\left|TN\right|}{\left|TP\right|+\left|TN\right|+\left|FP\right|+\left|FN\right|}$	The ratio of the number of correct predictions with respect to total observations
Precision (Urban et al. (2018), Zhang et al. (2016), Bandyopadhyay et al. (2013), Bedrikovetski et al. (2021)	$\frac{\left|TP\right|}{\left|TP\right|+\left|FP\right|}$	The ratio of the number of correct positive predictions with respect to total positive predictions
Recall/Sensitivity (Urban et al. (2018), Zhang et al. (2016), Bandyopadhyay et al. (2013), Bedrikovetski et al. (2021)	$\frac{\left|TP\right|}{\left|TP\right|+\left|FN\right|}$	The ratio of the number of correct positive predictions with respect to actual positive observations
F1 score/Dice-coefficient (Urban et al. (2018), Zhang et al. (2016), Bandyopadhyay et al. (2013), Bedrikovetski et al. (2021)	$2\times \frac{Recall\times Precision}{Recall+Precision}$	F1 score is the harmonic mean of both precision and recall

### 3.2 Evaluation of classifier performance

**FIGURE 3:**
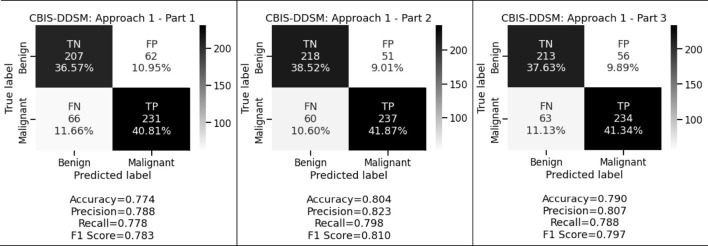
Confusion matrices of shallow network for CBIS-DDSM dataset.

**FIGURE 4:**
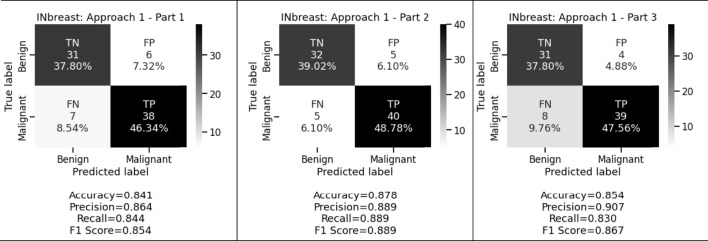
Confusion matrices of shallow network for INbreast dataset.

Figure 3 and Figure 4 display the performance of the shallow convolutional neural network in various scenarios on the CBIS-DDSM and INbreast datasets. For breast cancer detection, our proposed comparison reflects that experiment on the shallow network in the first aspect where only a two convolution layer-based model is proposed which obtained 77.4%, 78.8%, and 77.8% accuracy, precision, and recall for the CBIS-DDSM dataset, and 84.1%, 86.4%, and 84.4% accuracy, precision, and recall for INbreast dataset respectively; for the second aspect where to reduce the impact of overfitting, dropout strategy has been taken into account has obtained 80.4%, 82.3%, and 79.8% accuracy, precision, and recall for CBIS-DDSM dataset, and 87.8%, 88.9%, and 88.9% accuracy, precision, and recall for INbreast dataset respectively; and for the third aspect where again to reduce the effect of overfitting due to less number of images, augmentation technique has been applied which resulted into 79.0%, 80.7%, and 78.8% accuracy, precision, and recall for CBIS-DDSM dataset, and 85.4%, 90.7%, and 83.0% accuracy, precision, and recall for INbreast dataset respectively.

**FIGURE 5:**
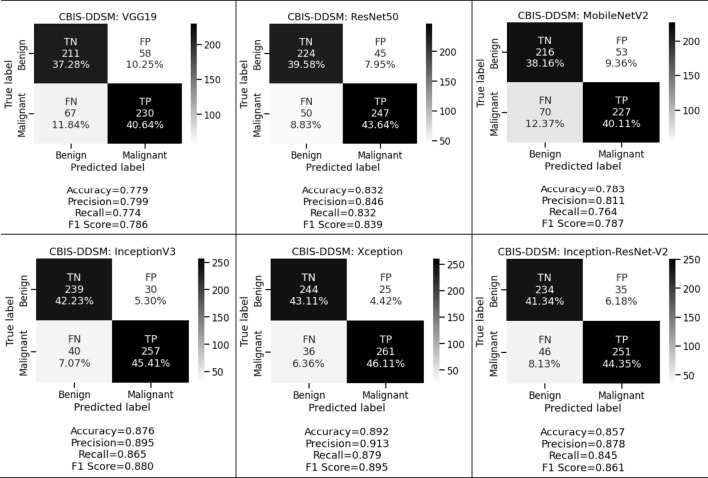
Confusion matrices of pre-trained CNNs for the CBIS-DDSM dataset.

**FIGURE 6:**
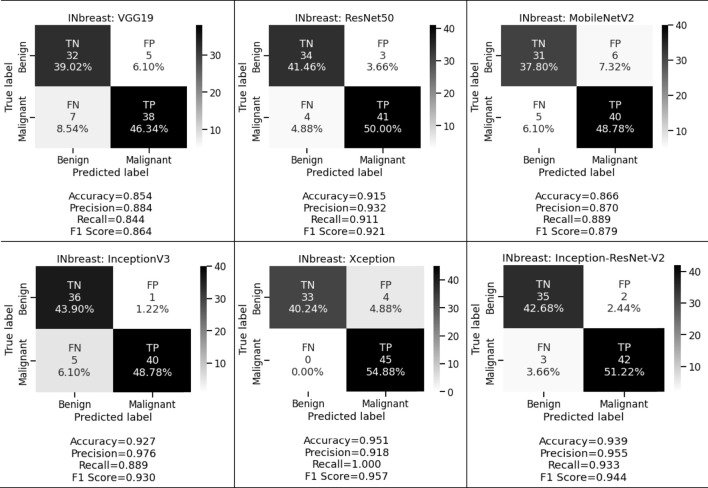
Confusion matrices of pre-trained CNNs for INbreast dataset.

Figure 5 and Figure 6 display the performance of the pre-trained deep convolutional neural networks on the CBIS-DDSM and INbreast datasets. We have also analyzed the other aspect where pre-trained deep convolutional neural networks have been executed with the help of transfer learning *via* fine-tuning on VGG19, ResNet50, MobileNetV2, InceptionV3, Xception, and Inception-ResNet-V2 respectively. From the above-mentioned experimental analysis on the CBIS-DDSM dataset, we obtained 77.9%, 79.9%, and 77.4% accuracy, precision, and recall for VGG19; 83.2%, 84.6%, and 83.2% accuracy, precision, and recall for ResNet50; 78.3%, 81.1%, and 76.4% accuracy, precision, and recall for MobileNetV2; 87.6%, 89.5%, and 86.5% accuracy, precision, and recall for InceptionV3; 89.2%, 91.3%, and 87.9% accuracy, precision, and recall for Xception, and 85.7%, 87.8%, and 84.5% accuracy, precision, and recall for Inception-ResNet-V2 respectively.

When the same fine-tuning-based experiment is performed on the INbreast dataset, we obtained 85.4%, 88.4%, and 84.4% accuracy, precision, and recall for VGG19; 91.5%, 93.2%, and 91.1% accuracy, precision, and recall for ResNet50; 86.6%, 87.0%, and 88.9% accuracy, precision, and recall for MobileNetV2; 92.7%, 97.6%, and 88.9% accuracy, precision, and recall for InceptionV3; 95.1%, 91.8%, and 100.0% accuracy, precision, and recall for Xception, and 93.9%, 95.5%, and 93.3% accuracy, precision, and recall for Inception-ResNet-V2 respectively. These observed results indicated that the proposed deep CNN with fine-tuning-based approach performed better than the shallow classifier in various conditions for both classification tasks.

We want to keep both the FP and FN low since they are essential to achieving the work’s purpose. First, our suggested system warns that patients with malignant cases who are mistakenly classified as benign instances may result in misclassification marked as a false negative (FN). Another possibility is that people who are diagnosed with cancer but do not have it might result in a false positive (FP). Both FNs and FPs have a large effect on misclassification, which results in incorrect diagnosis and poses health risks to people. We prioritized FP and FN equally by taking into account the F1 score and other performance assessment metrics. We observed that in the first approach we obtained 78.3%, 81.0%, and 79.7% for CBIS-DDSM and 85.4%, 88.9%, and 86.7% for the INbreast dataset respectively. Whereas in the second approach for the CBIS-DDSM dataset, we obtained 78.6%, 83.9%, 78.7%, 88.0%, 89.5% and 86.1% for VGG19, ResNet50, MobileNetV2, InceptionV3, Xception, and Inception-ResNet-V2 respectively. For the INbreast dataset, we obtained 86.4%, 92.1%, 87.9%, 93.0%, 95.7% and 94.4% for VGG19, ResNet50, MobileNetV2, InceptionV3, Xception, and Inception-ResNet-V2 respectively. Our proposed fine-tuning method for the Xception model gives the highest F1 score of 89.5% and 95.7% for breast cancer detection. Experimental results in terms of precision, recall, and F1 score conveys that the proposed model has a negligible rate of misclassification.

### 3.3 Discussion

According to the findings, the fine-tuned Xception classifier is a reliable model for the detection of breast cancer. The difference in the performances amongst the models because of the selected hyperparameters for each model which are learning rate, number of layers added after the last max-pooling layer of each model, number of hidden units for each layer added in fully-connected layers, and dropout rate respectively. The fine-tuned parameters are described in Section 2.2, Section 2.3, and Table 3 for reference. The main element influencing the outcome of the performances for the CBIS-DDSM and INbreast datasets may be due to the sharp fall in dimensions. The INbreast dataset has higher-quality mammograms as compared to the CBIS-DDSM dataset. The produced feature’s ability to discriminate is destroyed by dimensions that are too small. When deep-learning-based features are applied, the overfitting issue also appears in the INbreast dataset. Despite the lack of training samples in the INbreast dataset, the overfitting issue is not as severe as it is in the CBIS-DDSM dataset. This may be mostly due to the excellent mammograms in the INbreast dataset. Along with that because of the fine-tuned parameters, all models performed much better for the INbreast dataset even with lesser sample size. For this work, rigorous experiments have been carried out, such as when the models were tested on the INbreast dataset after being trained on the CBIS-DDSM dataset and *vice versa*. Obtained results were not promising compared to the results obtained when trained and tested on the same dataset, but it will lead to the future scope of research. Though the suggested strategy performs better, this study does not take into account other clinical data, such as other medical problems, geographic location, etc. Future research that takes these parameters into account might enhance computer-aided methods for early breast cancer diagnosis and tailored medicine treatment. The transfer-learned features are more reflective of the natural image properties and may not always represent the delicate qualities of medical images since the vast dataset used for transfer learning incorporates natural images. As a result, it is anticipated that transfer learning from a big dataset in the same domain would result in the creation of a breast cancer detection system that is more effective. The proposed models have been put through a performance comparison with cutting-edge methods. Table 5 compares the performance of our suggested classifiers on the CBIS-DDSM dataset as well as the INbreast dataset. These outcomes demonstrate that our suggested fine-tuned Xception classifier outperforms other classifiers.

**TABLE 5:** Comparison of the proposed approach with the state-of-the-art techniques.

Dataset	Author	Year	Accuracy (%)
CBIS-DDSM	Shams et al. (2018)	2018	75
CBIS-DDSM	Tsochatzidis et al. (2019)	2019	89
CBIS-DDSM	Falconi et al. (2020)	2020	84.4
CBIS-DDSM	Ansar et al. (2020)	2020	74.5
CBIS-DDSM	Zhang et al. (2020)	2020	87.05
CBIS-DDSM	Proposed (Xception classifier with fine-tuning)	2022	**89.2**
INbreast	Dhungel et al. (2017)	2017	90
INbreast	Carneiro et al. (2017)	2017	90
INbreast	Shi et al. (2019)	2019	83.9
INbreast	Zhang et al. (2020)	2020	87.93
INbreast	El Houby and Yassin. (2021)	2021	93.04
INbreast	Proposed (Xception classifier with fine-tuning)	2022	**95.1**

Bold values are the significant results achieved for the proposed work.

## 4 Conclusion

In this article, we compared shallow convolutional neural networks to deep convolutional neural networks and introduced pre-trained models that have been fine-tuned to classify full-mammogram images as benign or malignant. ROI-based mammography images can produce better outcomes, but grinding up a sufficient degree of precision for full-mammogram images is a laborious process. Our suggested trained CNNs can pick up various information included in individual images. The improved CNN models produce a more effective image categorization method than the individual CNNs that are trained from scratch. Due to Xception’s optimization of ResNet, which allows it to inherit not just ResNet’s benefit of residual connection but also its capability to extract objects when occluded by occlusions using depth-wise separable convolution, it obtains the greatest performance when extracting features. When compared to the other classifiers, the comparison analysis shows that the performance of the improved Xception classifier is quite significant. As a result, among all of our suggested methods, the improved Xception classifier performs the best at detecting breast cancer, with acceptable levels for all performance metrics in the range of .87–.91 for the CBIS-DDSM dataset and .91 to 1.00 for the INbreast dataset respectively.

## Data Availability

Publicly available datasets were analyzed in this study. This data can be found here: CBIS-DDSM: https://wiki.cancerimagingarchive.net/pages/viewpage.action?pageId=22516629 INbreast: https://www.kaggle.com/datasets/ramanathansp20/inbreast-dataset.
